# Supplementary material for: Kinetics of Drug Molecule Interactions with a Newly Developed Nano-Gold-Modified Spike Protein Electrochemical Receptor Sensor
Source: Biosensors (Basel). 2022 Oct 17;12(10):888. doi: 10.3390/bios12100888 (PMC9599096; doi:10.3390/bios12100888)
Supplement: Supplementary file 1 [file biosensors-12-00888-s001.zip › biosensors-1948378-supplementary-done.pdf]

biosensors

MDPI

SUPPLEMENTARY DATA

Kinetics of Drug Molecule Interactions with a Newly Developed Nano-Gold-Modified Spike Protein Electrochemical Receptor Sensor

Preparation and Characterization of AuNPs

The preparation of gold nanoparticle sol adopts sodium citrate reduction method of chloroauric acid. The specific operation is as follows: the sodium citrate solution and neutral chloroauric acid solution are mixed in a volume ratio of 1:25, and the pH of the solution is adjusted with potassium carbonate and sodium carbonate. The value is 7.0, then put the solution in a microwave oven, heat it with medium heat for 10-15 minutes, observe the color change of the solution, stop heating when the solution turns into a bright wine red, indicating that the nano-gold particle sol was successfully prepared. The Erlenmeyer flasks were then wrapped in foil and stored in the dark at 4°C. The AuNPs were characterized using UV-Vis spectrophotometer and Transmission Electron Microscope (TEM).

The synthesized AuNPs in this study were bright red wine in color. The scanning spectra in the wavelength range of 400–700 nm are shown in Fig. 1. The result showed a strong absorption peak at 521 nm, roughly indicating that the average particle size of AuNPs was 15–20 nm. The TEM results of AuNPs AuNPs-HRP and AuNPs-HRP/spike protein are shown in Fig. 1A,B, C and D. The figure shows that the synthesized AuNPs in this study had a regular shape, uniform particle size, and average particle size of about 15 nm with no aggregation. The characterization of AuNPs using UV–Vis spectra was the same as that using TEM scanning, suggesting that the particle size of AuNPs was 15–20 nm, which could be used for subsequent experiments. From Fig 1C, it can be seen that the nanogold and HRP are cross-linked together in a pine tree shape, and from Fig 1D, it can be seen that the spike and AuNPs-HRP binding in a dendritic fashion.

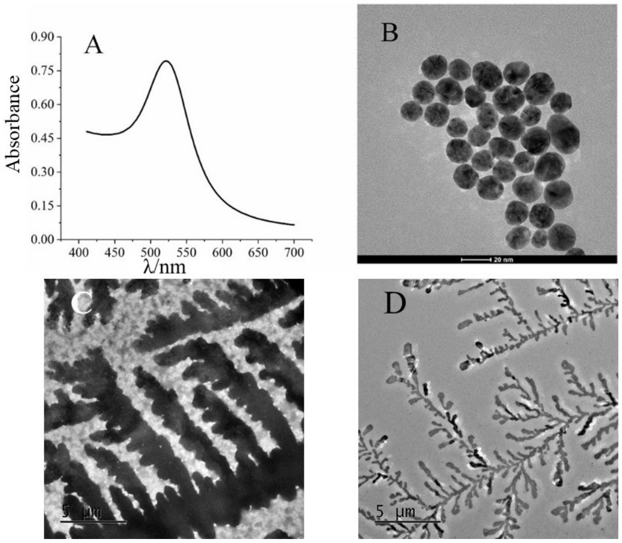

Figure S1. (A) Spectral absorption curve of AuNPs; (B) TEM of AuNPs; (C) TEM of Thi-Chit/AuNPs-HRP; (D) TEM of AuNPs-HRP/spike protein.

Table S1. The affinity value of the optimal spike protein/chloroquine diphosphate binding conformation.

Mode	Affinity (kcal/mol)	Dist from Rmsd l.b.	Best Mode rmsd u.b.
1	-5.3	0.000	0.000
2	-5.1	3.482	5.792
3	-4.9	22.403	24.844
4	-4.7	20.594	23.460
5	-4.7	5.012	9.230
6	-4.6	42.186	44.168
7	-4.4	4.498	8.498
8	-4.2	31.336	33.803
9	-4.2	19.573	21.482

Table S2. The affinity value of the optimal spike protein/hydroxychloroquine binding conformation.

Mode	Affinity (kcal/mol)	Dist from rmsd l.b.	Best Mode rmsd u.b.
1	-6.5	0.000	0.000
2	-6.4	2.976	4.370
3	-6.2	36.597	39.011
4	-6.2	37.606	39.833
5	-6.1	2.568	3.894
6	-5.9	36.353	38.752
7	-5.9	2.761	4.579
8	-5.9	35.418	37.908
9	-5.7	15.599	18.113

Table S3. The affinity value of the optimal spike protein/ribavirin binding conformation.

Mode	Affinity (kcal/mol)	Dist from rmsd l.b.	Best Mode rmsd u.b.
1	-7.0	0.000	0.000
2	-6.8	2.467	3.421
3	-6.5	2.963	5.174
4	-6.4	3.321	3.967
5	-6.4	2.939	5.052
6	-6.3	24.005	25.251
7	-6.3	2.801	5.554
8	-6.2	38.417	39.564
9	-6.1	3.318	5.075

Table S4. The affinity value of the optimal spike protein/bromhexine binding conformation.

Mode	Affinity (kcal/mol)	Dist from rmsd l.b.	Best Mode rmsd u.b.
1	-7.3	0.000	0.000
2	-7.2	1.556	2.156
3	-6.0	35.860	37.873
4	-5.8	22.137	25.538
5	-5.8	26.229	29.021
6	-5.4	7.785	10.327
7	-5.3	2.523	3.590
8	-5.3	10.909	13.432
9	-5.2	30.080	32.892

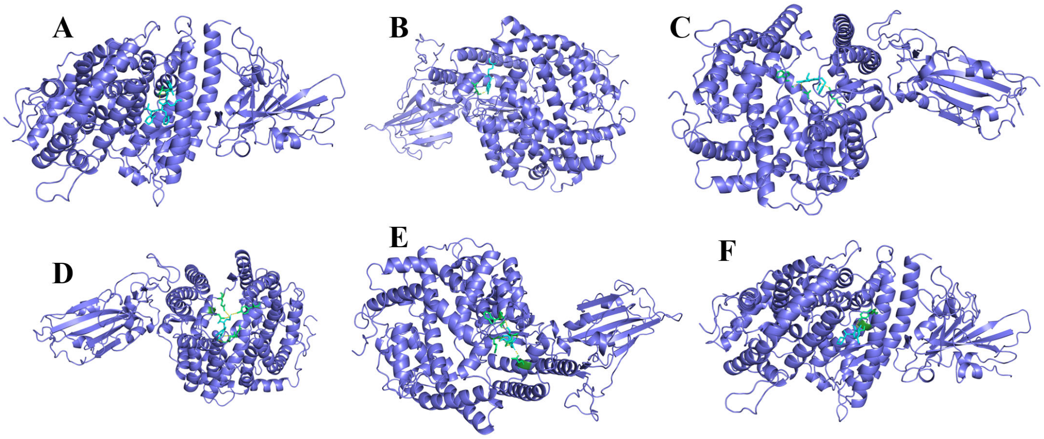

Figure S2. Crystal structure of spike protein and its binding site with drug molecules: (A) lopinavir, (B) chloroquine diphosphate, (C) abridol, (D) hydroxychloroquine, (E) ribavirin, (F) bromhexine.

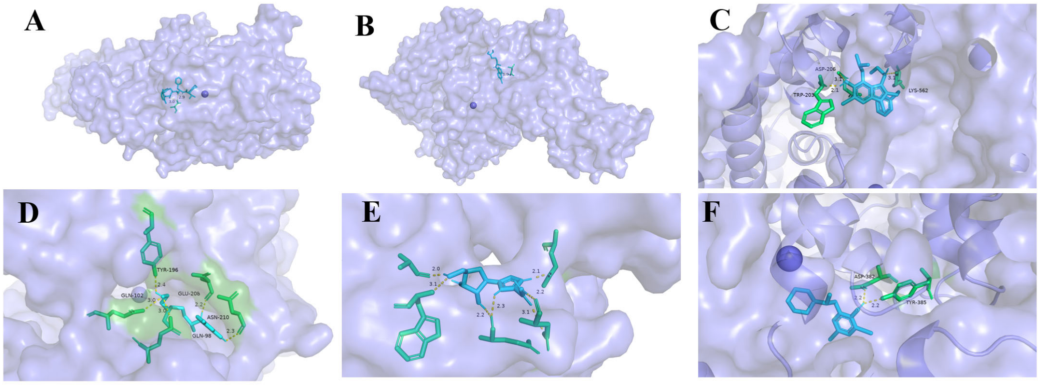

Figure S3. Three dimensional hydrogen bonding surface diagram of binding sites: (A) lopinavir, (B) chloroquine diphosphate, (C) abridol, (D) hydroxychloroquine, (E) ribavirin, (F) Bromhexine.

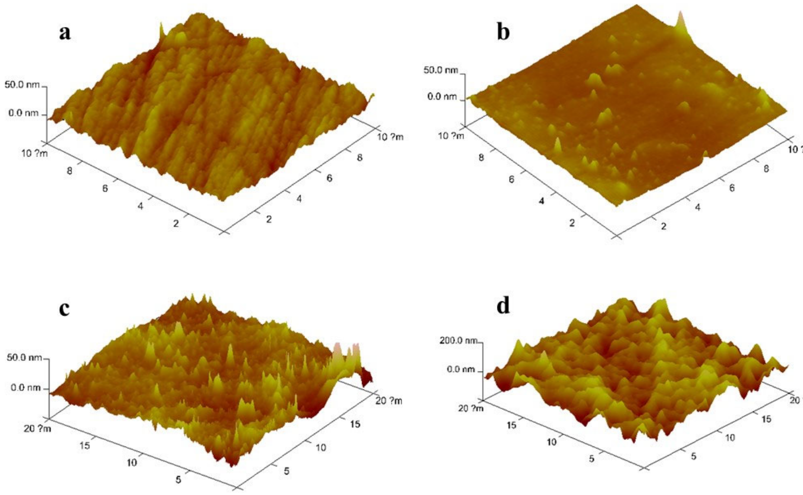

Figure S4. Atomic force microscopy characterization of electrodes during the modification process and surface morphology parameters of electrodes at different assembly stages: a to d in the order of GCE, assembled thi-chit, assembled AuNPs-HRP, assembled spike protein.

As shown in Fig.S4, compared with the bare electrode, the roughness Ra decreased (3.31 to 0.423 nm) after the film was dried and formed by dropping the sulfur thi-chit, which is because the film-forming chitosan covered the scratches on the surface of the bare electrode, and the peak height also decreased from 99.1 nm to 2.57 nm; Ra increased (0.423 to 7.17 nm) after the adsorption of AuNPs-HRP, which is because the nanogold is in spherical shape, uniformly covering the smooth membrane surface will obviously make Ra increase, while the peak height will also increase, 2.57 rose to 99.4 nm; Ra slightly decreased after assembling spike protein, Au particles adsorbed a large number of proteins through Au-S, which covered the gaps between Nano-Au particles, and the peak height also slightly decreased at this time.
